# Diagnostic imaging findings and management of osteoarthritis in pigs with image‐guided intra‐articular injections

**DOI:** 10.1111/vsu.70002

**Published:** 2025-08-27

**Authors:** Reed H. Schultz, Jan F. Hawkins, Jesus A. Hermida

**Affiliations:** ^1^ Department of Veterinary Clinical Sciences, College of Veterinary Medicine Purdue University West Lafayette Indiana USA; ^2^ Present address: College of Veterinary Medicine North Carolina State University Raleigh USA

## Abstract

**Objective:**

To describe the clinical and diagnostic imaging findings of pet pigs with osteoarthritis and determine the efficacy of intra‐articular (IA) injections in managing lameness.

**Study design:**

Retrospective observational study.

**Animals:**

A total of 16 pet pigs diagnosed with osteoarthritis and treated with non‐steroidal medications and IA injections.

**Methods:**

Cases, diagnostic imaging, and clinical data were collected from the medical records of Purdue University. The owners were interviewed by telephone to collect follow‐up data regarding the degree of lameness following joint treatment.

**Results:**

A total of 13 of 16 pigs presented for lameness evaluation. A diagnosis of osteoarthritis was determined with computed tomography (CT) (*n* = 10) or digital radiography (*n* = 7). In all, 24 joints were treated with triamcinolone. Following injection, 13 of 16 (81%) pigs showed improvement in lameness, as measured by decreases in lameness scores assigned by the owner (*p* = .0183). The duration of the effect ranged from a few weeks to several months. Those with no or minimal improvement tended to have advanced osteoarthritis in joints other than those treated. There were no complications reported. The use of CT for injection guidance did not show a significant difference in outcomes over the use of radiographic guidance.

**Conclusion:**

With an accurate diagnosis, IA injections seemed to be a beneficial therapy for pet pigs.

**Clinical relevance:**

The use of IA corticosteroids to treat lameness due to osteoarthritis in pigs represents an effective therapy that reduces lameness.

## 1 INTRODUCTION

Osteoarthritis is a common cause of lameness in pigs, especially those maintained as pets or in farm animal sanctuaries. Additional causes of lameness may include osteochondrosis and septic arthritis, which are well described in commercial pigs, but literature regarding pet pigs is lacking.^1^, ^2^ Regardless of the cause, osteoarthritis leads to signs of pain and reduced mobility. Previously, osteoarthritis in pigs was managed with nonsteroidal anti‐inflammatory drugs (NSAIDs) or, when unresponsive to conservative management, euthanasia or shipment for slaughter was frequently chosen.^3^ With the rising popularity of pet pigs and farm animal sanctuaries, treatment options are being sought to improve the quality of life of pigs with osteoarthritis.

Management tools used at our institution have included the combined treatment of NSAIDs and intra‐articular (IA) injections. Intra‐articular medications used have included corticosteroids, hyaluronic acid, and polyacrylamide gel. It has also been standard to perform these injections with imaging guidance, either radiographs or computed tomography (CT). Pigs can demonstrate variable body morphologies and the sites for IA injections are not described so image guidance ensures accurate delivery of the medication. Computed tomography also improves the accuracy of diagnosis as commonly affected joints such as the elbow have significant superimposition from the thorax in some radiographic projections.

IA steroids, such as triamcinolone and methylprednisolone, have been widely used in veterinary medicine, particularly in horses.^4^ The use of IA steroids in pigs has been studied as an experimental model for human joint disease,^5^ but the long‐term effects of these treatments have not been reported in pet pigs. This was a retrospective observational study of pet or sanctuary‐housed pigs that were treated for osteoarthritis using IA injections. The objective was to characterize the outcomes of pigs that received IA injections to manage osteoarthritis. We hypothesized that IA therapy would improve signs of lameness and result in improved mobility without adverse effects. A second hypothesis was that CT‐guided injections would result in improved lameness scores compared to joints injected with radiographic guidance.

## 2 MATERIALS AND METHODS

Medical records for all pigs admitted to the Purdue University Veterinary Hospital from January 2010 to June 2024 were searched for pigs admitted for lameness evaluation and treated with IA injections. Pigs were excluded from the study if they were diagnosed with a joint disease other than osteoarthritis. Information collected from the medical record included the patient signalment, clinical findings, diagnostic imaging performed, imaging findings and diagnosis, method of restraint (sedation vs. general anesthesia), joint(s) treated, medication and dose used, and use of image guidance for injection. Severity of osteoarthritis in the affected joints was graded as mild, moderate or severe based on the imaging findings. Multiplanar (MPR) and three‐dimensional (3D) reconstruction were performed using DICOM imaging analysis software (OsiriX; Bernex, Switzerland).

### 2.1 Client surveys

Institutional animal care and use committee approval was not required to conduct owner surveys for a retrospective study. Owners were contacted by telephone interview and surveyed regarding their opinions on the outcomes of the treatments (Supplemental file 1). Questions were asked regarding the response of the lameness following treatment, duration of improvement in lameness, concurrent medications given, complications, follow up IA injections, and overall satisfaction. Specifically, owners were asked to grade the scale of lameness from 1 (no lameness visible) to 5 (non‐weight bearing or reluctant to move) prior to and following injections.

### 2.2 Statistical analysis

Due to small sample sizes, summary statistics are reported as median and range. To compare the effects of clinical data, severity of disease, Mann–Whitney U tests were performed for continuous and ordinal variables, and Fisher's exact test was used for categorical data. A two‐way ANOVA test was used to compare the effects of the technique and the effect of injection using the lameness scores before and after injection. A *p*‐value < .05 was considered statistically significant. Data analysis was performed in a commercial statistical analysis software (Prism, GraphPad Software, Boston, Massachusetts).

## 3 RESULTS

### 3.1 Clinical data

During the study period, 58 pigs presented for evaluation of lameness or non‐weightbearing and there were 17 pigs that had lameness evaluation and received an IA injection. Of those receiving IA injections, one pig was excluded for a diagnosis of shoulder osteochondrosis without osteoarthritis. Of the remaining 16 pigs, there were 11 spayed females (3 were spayed at the same time as the injections), four castrated males, and one intact male. Breeds included Vietnamese Pot‐Bellied Pig (7), unknown (5), mixed breed (3), Yorkshire (1), and Kune Kune (1). The age at the time of injection ranged from 2.2 to 14.1 years (median: 6.9 years), and bodyweight ranged from 18 to 386 kg (median: 87.5 kg). Body condition scores were recorded for 15 of 16 pigs and ranged from 5.5 to 9 out of 9, with a median 7 out of 9. A total of 24 joints were injected. The most common joint injected was the elbow (20). There was one each of the shoulder, stifle, metacarpophalangeal joint and proximal interphalangeal joints injected. Bilateral elbow injections were performed in seven pigs, with one of those pigs receiving an injection of the right metacarpophalangeal joint.

The presenting complaint was lameness for 13 pigs, two of which presented a second opinion on treatment options for previously diagnosed osteoarthritis at other institutions. Two pigs presented for spay and had pre‐existing lameness, which prompted diagnostic imaging and treatment of the affected joints. One pig presented for dermal masses but also had a bilateral forelimb lameness, requiring further examination. One pig presented for lethargy, which was attributed to pain secondary to osteoarthritis, as whole‐body CT did not identify any other abnormalities. Two pigs had diagnostic imaging and injections performed on separate visits. Physical examination revealed left forelimb lameness in five pigs, right forelimb lameness in five pigs, bilateral forelimb lameness in two pigs, and right hindlimb and left hindlimb lameness in one pig each. The medical record did not specifically identify lameness in two pigs. Other findings included decreased or non‐weight bearing of the affected limb in four pigs and difficulty standing or walking in three pigs. Soft tissue swelling around the affected joint (elbow) was identified in one pig. All pigs were recommended to undergo diagnostic imaging with digital radiographs or computed tomography.

Medications given to the pigs at the time of injection were collected from the medical record when available and through the follow‐up questionnaire. At the time of injection, 13 of 16 pigs were on medications for lameness at the time of injection, including an NSAID (13), gabapentin (5), tramadol (1), and prednisone (1). Five pigs were on a combination of an NSAID and gabapentin and one of those pigs was also receiving tramadol and prednisone. Three pigs were on no medications at the time of injection, and no pigs were given additional medications following the joint injections.

### 3.2 Diagnostic imaging and treatment

**FIGURE 1:**
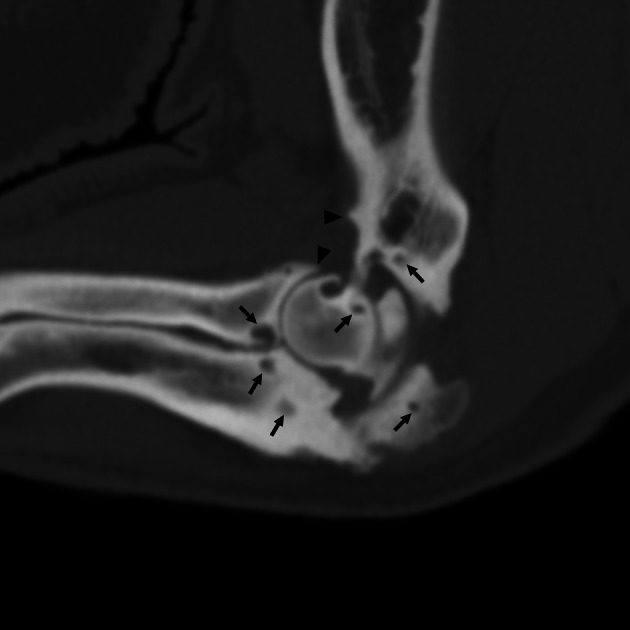
Sagittal multiplanar reconstruction of the left elbow of a pig with multifocal osseous cyst‐like lesions (arrows) in the humeral condyle, radial head and ulna as well as periarticular osteophytes (arrowheads).

**FIGURE 2:**
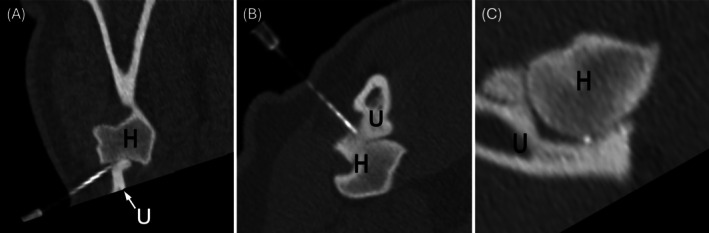
Multiplanar reconstruction views of computed tomography‐guided injection of the right elbow with a lateral approach between the ulna (U) and the humeral condyle (H). Dorsal is to the top, lateral to the left, and cranial to the left. The viewing axes are aligned with the needle for ease of visualization and do not represent standard projections.

Computed tomography was used for diagnosis in 10 cases, and radiographs were used for diagnosis of lameness in seven cases; one pig underwent unilateral elbow radiographs prior to CT and bilateral elbow injections. Amongst the joints that were treated, the most common diagnostic imaging findings included osteophytes (*n* = 22/24 [92%]), osseous cyst‐like lesions (*n* = 8/24 [31%]) and IA fragments or mineralization (*n* = 8/24 [31%]) (Table 1, Figure 1). The severity of arthritis was graded as mild in five joints, moderate in five joints and severe in 14 joints. Mild and moderate osteoarthritis were diagnosis in joints other than the treated joints in five pigs and one pig, respectively. Imaging guidance for IA injection was performed using radiographs in 10 cases and CT in six cases (Figure 2).

**TABLE 1:** Summary of radiographic and CT findings of the treated joints.

Radiographs		Computed tomography			
Imaging finding	Frequency	Imaging finding	Frequency		
Elbow (*n* = 5)		Elbow (*n* = 16)			
Distal humerus osteophytes	4 (80%)	Radial osteophytes	14 (88%)		
Radial head osteophytes	5 (100%)	Humeral osteophytes	13 (81%)		
Radial head lucency, cyst‐like lesion	1 (20%)	Ulnar osteophytes	13 (81%)		
Triceps enthesophyte	1 (20%)	Intra articular osseous body/fragment	6 (38%)		
Extracapsular mineralization	1 (20%)	Incomplete ossification of the humeral condyle	5 (31%)		
Radial head sclerosis	1 (20%)	Subchondral cystic lesions, radius and humerus	4 (25%)		
Widened joint space	2 (40%)	Subchondral cystic lesions, radius, humerus and ulna	3 (19%)		
Stifle (*n* = 1)		Subchondral cystic lesions, radial head only	1 (6%)		
Intracapsular swelling	1 (100%)	Subchondral bone sclerosis	4 (25%)		
Patellar enthesophyte	1 (100%)	Fluid distension	2 (13%)		
Intra‐articular mineralization	1 (100%)	Triceps enthesopathy	2 (13%)	
Trochlear osteophytes	1 (100%)	Narrowed joint space	1 (6%)	
Proximal tibia osteophytes	1 (100%)	MCP (*n* = 1)			
Shoulder (*n* = 1)		Periarticular osteophytes	1 (100%)	
Blunting of the greater tubercle	1 (100%)	PIP (*n* = 1)			
Humeral head osteophytes	1 (100%)	Irregular joint space	1 (100%)		
Glenoid sclerosis	1 (100%)	Periosteal proliferation	1 (100%)		
Mineralization	1 (100%)	Periarticular bone formation	1 (100%)

*Note*: One elbow of a pig that received bilateral IA injections is represented in both the radiograph and computed tomography groups.

Abbreviations: CT, computed tomography; IA, intra‐articular; MCP, metacarpophalangeal joint; PIP, proximal interphalangeal joint.

**FIGURE 3:**
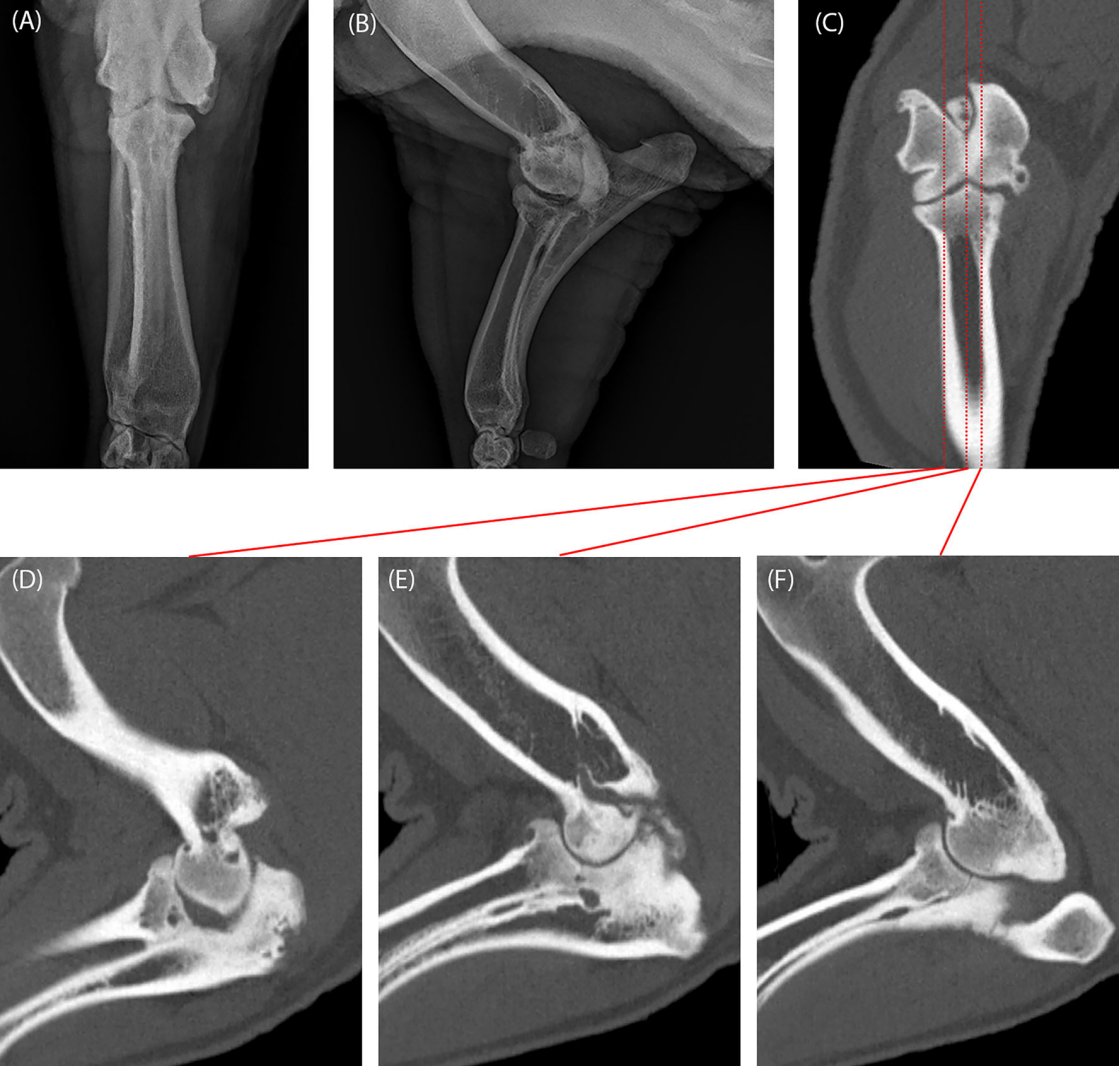
(A) Cranial‐caudal and (B) lateral‐medial radiographic views of a pig with severe degenerative joint disease that also underwent computed tomography during the same visit. The transverse multiplanar reconstruction (C) shows the location of three different sagittal reconstructions presented from lateral to medial (D–F). Computed tomography provides significant improvement in detection of osseous cystic lesions and sclerosis throughout the joint, as well as complete evaluation of the joint space.

One 5‐year‐old Vietnamese Pot‐Bellied pig had radiography of the right elbow performed prior to CT of both forelimbs (Figure 3). Cranial‐caudal and medial‐lateral digital radiographs identified a well‐defined ovoid focus of reduced mineral opacity with a sclerotic rim in the proximolateral radius, smaller foci in the caudoproximal radius and ulna, osteophytes at the humeral condyle, epicondyles, and cranioproximal radius, and a small enthesophyte at the triceps brachii tendon, with a radiographic diagnosis of moderate osteoarthritis. Computed tomography of the same elbow detected chronic incomplete humeral condyle ossification, and multifocal to coalescing hypoattenuating foci and osteophytes along the ulnar trochlear notch, which radiography missed. Computed tomography also provided improved imaging of the joint space, which was markedly narrowed in the lateral aspect of the radio‐humeral joint.

All pigs that received an IA injection were injected with triamcinolone (Kenalog, 10 mg/mL), and six pigs had the amount administered recorded in the medical record with a range of 5 to 15 mg per joint (*n* = 6, median = 6 mg) and a total body dose of 0.10 to 0.55 mg/kg (*n* = 6, median = 0.27 mg/kg). In addition to triamcinolone, two pigs each received either hyaluronic acid (Hyalovet, 20 mg per joint) or polyacrylamide gel (Noltrex, 4%, 1 syringe per joint). The IA injections were performed under inhalant general anesthesia (*n* = 13/16 [81%]), injectable sedation (*n* = 2/16 [13%]), or injectable general anesthesia (*n* = 1/16 [7%]). For 14 pigs, there was no attempt to collect synovial fluid prior to injection. Synovial fluid was collected from two pigs prior to joint injections. One pig that received bilateral elbow injections also had bilateral chronic scapulohumeral joint luxation and pseudarthrosis. At the same time as the injections, the left shoulder underwent surgical arthrodesis with transarticular screws, but surgery could not be performed on the left shoulder due to anesthesia concerns.

### 3.3 Synovial fluid analysis

**FIGURE 4:**
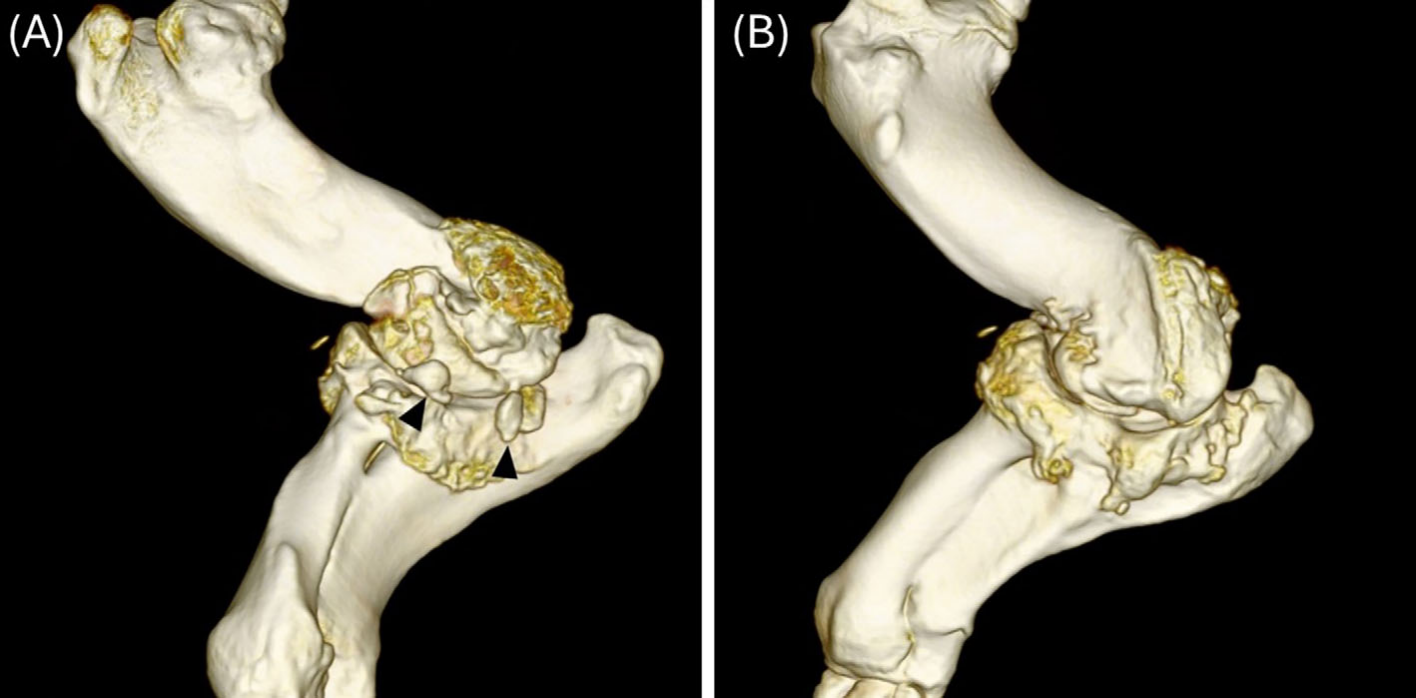
(A) Lateral and (B) medial view of a three‐dimensional reconstruction of a left elbow in a pig with severe osteophytosis of the humerus, radius and ulna as well as large intra‐articular fragments (arrowheads). Cranial is to the left. Synovial fluid analysis of this joint revealed severe neutrophilic inflammation.

Two pigs had samples of synovial fluid from both elbows submitted for fluid analysis and cytology at the time of injection; one was during the initial injection, and one was during a follow‐up injection. In pig 1, the synovial fluid seen during injection was cloudy, prompting collection and analysis. The synovial samples had a total protein of 5.7 and 6.2 mg/dL (<3.8 g/dL^6^), and both joints had an estimated cell count of approximately 100 000 cells/μL (<5000 cells/μL^6^), but an automated cell count could not be performed. The cells were composed mostly of non‐degenerate neutrophils (99%), and a diagnosis of severe neutrophilic inflammation was made. A culture performed on the same sample did not yield any growth. Computed tomographic findings in this pig included severe periarticular osteophytes and multifocal small lucencies within the distal humerus and proximal radius (Figure 4). While a septic process cannot be excluded, even with negative culture results, the CT findings were similar to previous pigs at this institution, so the joint was injected with steroids during the same anesthetic event. Following the injections, the owner reported that this pig improved significantly and was still improved at follow up, 10 months after the injection.

Fluid analysis was performed on synovial fluid from both elbows of a second pig 25 months after its initial treatment. The sample was collected due to the increased volume of fluid but was not intended to be submitted for analysis, according to the clinician who performed the injection (JAH, personal communication). This pig was also receiving IA injections from its referring veterinarian every 3–4 months but were reportedly having less efficacy. The synovial fluid analyses were of low cellularity (no cell count reported) with a protein of 2.5 and 3.2 g/dL (<3.8 g/dL^6^). The cells were almost entirely large mononuclear cells with rare non‐degenerate neutrophils.

### 3.4 Follow‐up

Follow‐up information was obtained from all owners by telephone. Duration of follow‐up ranged from 7 to 52 months (median: 26.1 months). Owners reported marked improvement in lameness in eight pigs, slight improvement in five pigs, and no difference in three pigs. No lameness worsened following the injections, according to owners. Amongst pigs in which improvement was seen, and owners were able to recall, the onset of effects were reported as less than 1 week (3), about 1 week (4), and 2 weeks (1). The peak effect of the injections was reported as 2 weeks (5), 1 month (1), 2 months (1), 3 months (1), and 6 months (1). The duration of the effect ranged from 2 weeks to several months. A total of 11 clients reported that they noticed an increase in activity following the IA injections. Six of the 13 pigs were able to have at least one medication decreased or discontinued following the injection due to improvements in lameness. Client satisfaction with the use of IA injections in their pigs was reported as satisfied in 10/16 cases, slightly satisfied in 1/16 cases, neutral in 4/16 cases, and dissatisfied in 1/16 cases. All but one client would recommend IA injections to other owners if indicated. This client owned the pig that also had shoulder luxation and underwent arthrodesis of one shoulder, which was euthanized 8 months after the treatments due to mobility concerns. No clients reported any adverse reactions or complications following the injections.

Four of 16 pigs returned for a follow‐up visit, ranging from 41 to 762 days (median: 140 days) after the IA injection. One pig presented for a new problem (squamous cell carcinoma of the ear). Three pigs presented for recurrence of lameness and received joint injections in the same joint treated previously, ranging from 41 to 762 days (median: 238 days). All owners reported improvement in lameness following injection, but the lameness returned shortly before the follow‐up visit. There was not enough information in the medical record to compare the degree of lameness at the recheck visit compared to the initial visit.

### 3.5 Lameness scoring

**FIGURE 5:**
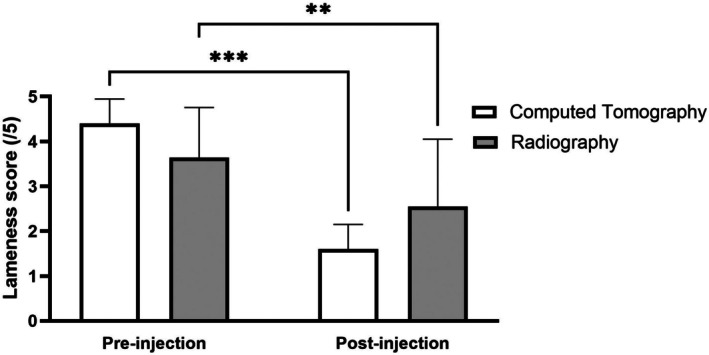
Reduction in lameness scores as reported by the owners before and after intra‐articular injection using computed tomography or radiography for guidance. Significant reductions in lameness were seen in both groups as shown by the asterisks (*p* < .05).

**FIGURE 6:**
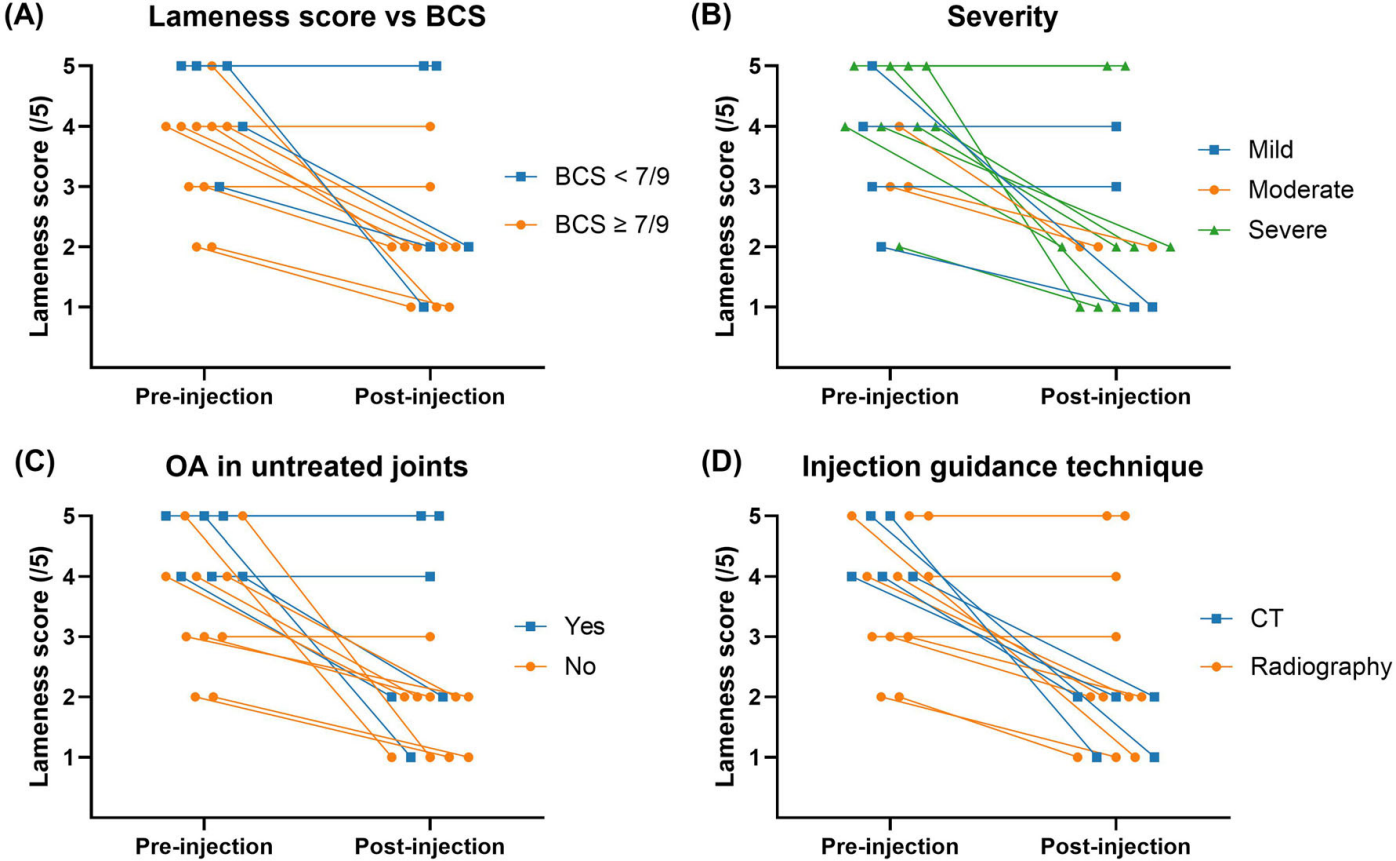
Dot plot representations of the reduction in lameness scores of all pigs categorized by (A) body condition score, (B) disease severity of the treated joint, (C) presence of diagnosed osteoarthritis in untreated joints and (D) the imaging technique used for injection guidance. No groups displayed a statistically significant difference.

Clients were asked to grade the observed lameness at the time of the injection and at the peak effect following the injection. Amongst all pigs, there was a decrease in median lameness scores from 4 to 2 which was statistically significant (*p* = .0183) (Figure 5). To analyze the difference between CT‐ and radiograph‐guided techniques, two‐way ANOVA was used to compare the pre‐ and post‐treatment lameness scores. The difference in lameness scores was not significantly different between the groups (*p* = .8681). There was no difference between the CT‐guided and radiograph‐guided groups when comparing weight, sex, and preinjection lameness score. There was also no significant difference in response to treatment when comparing BCS (<7/9 vs. ≥7/9), severity (mild, moderate or severe), or whether there was diagnosed OA in untreated joints (Figure 6).

## 4 DISCUSSION

Most pigs in this study showed a significant reduction in lameness scores following IA injection, with 13 out of 16 pigs demonstrating improvement in lameness following the injections, according to the owners (Figure 4). This supported our first hypothesis that IA injections would lead to improvements in lameness. For the pigs that showed no improvement, they commonly had arthritis in joints aside from those injected, which may explain the poor response; however, it was typically mild to moderate. While this was not a statistically significant result, three of the four pigs that showed no decrease in lameness scores had OA diagnosed in other joints. Many factors may have contributed to the lack of a positive response, including the severity of joint disease, the number of joints affected, and inaccurate administration of the medication. Three‐dimensional diagnostic imaging greatly improves the ability to assess all aspects of any affected joints and soft tissue structures, especially in pigs whose anatomy and behavior are typically not amenable to thorough physical and lameness examinations and high‐quality radiographs. At our clinic, the lameness examinations are based on observation of the gait at a walk in a small area, and the pigs typically have considerable lameness, which warrants investigation with diagnostic imaging. While sedated or anesthetized for diagnostic imaging, palpation and manipulation of the limbs is performed to assess the joints for effusion, joint capsule thickening, and decreased range of motion. Due to these challenges, it has become routine at our clinic to recommend CT for all pigs presenting for lameness.

There was no significant difference between pigs injected using CT or radiographs for imaging guidance (*p* = .8681). Therefore, our second hypothesis was rejected. Regardless of the imaging modality used, the authors recommend that imaging guidance is always used for IA injections in pet pigs as they are commonly overweight with severe joint pathology, which makes the palpation of landmarks for IA injection difficult. However, the improved diagnostic quality and ability to assess the entire patient, if indicated, is likely beneficial in providing the most appropriate therapy.

The elbow was the most common joint affected and subsequently treated, and this is supported by previous literature investigating causes of joint disease in production pigs at slaughter.^7^ In that study, nearly all diseased joints were classified as having lesions consistent with osteochondrosis. Osteochondrosis is a common problem associated with fast‐growing pigs in a production setting, but whether osteochondrosis is caused by or a result of higher growth rates cannot be determined.^2^ In our population, the presence of cystic lesions in the elbow was always associated with moderate to severe osteophytosis. Given the prevalence of cystic lesions in association with osteoarthritis in several pigs, it is plausible that osteochondrosis may precede arthritis, or that cystic lesions develop as a pathologic change of arthritis. Incomplete ossification of the humeral condyles and subchondral bone cystic lesions have been previously described as common findings in pigs with osteoarthritis and have been correlated to lameness with an odds ratio of 9.89 and 9.59, respectively.^8^ Additionally, pigs, especially pot‐bellied pigs, tend to exhibit outward rotation of the forelimbs, which may place abnormal biomechanical stresses on the joints, which may further the progression of osteoarthritis. Finally, many of the affected pigs are overweight with minimal activity levels, which leads to further abnormal joint forces contributing to osteoarthritis.

The duration of effect seen by improvements in lameness was seen to be wide ranging, from a few weeks to well over 1 year. These results seem similar to those in horses where the use of triamcinolone is routine for the treatment of osteoarthritis. Triamcinolone is describe as a medium length duration steroid compared to other steroids used for IA injections^9^ and one study showed that about half of horses are back in their previous level of work at 3 months indicating a similar duration of effect seen in this study.^10^ Due to small sample sizes, it is not possible to determine which factors may have contributed to the wide range in duration of action for this group.

While no complications were reported in this study, potential risks of IA injections, mainly joint infection, have been documented in other species so it is reasonable to expect these complications to be a risk of IA injections in pigs.^9^ IA injections should always be performed with strict aseptic technique to minimize this risk. The use of corticosteroids and other medications in pet pigs is off‐label, and veterinarians should comply with the Animal Medicinal Drug Use Clarification Act (AMDUCA), even for pet pigs, as they are legally considered food animals. Consultation with the Food Animal Residue Avoidance Databank (FARAD) is recommended regardless of the intended use of the pig.

The synovial fluid analysis conducted on two pigs provided limited insight into the inflammatory processes within the joints before and after treatment. At the time of the injection in pig 1, the cloudy synovial was collected and based on the abnormal synovial fluid appearance, it was submitted for analysis. The imaging findings of decreased joint space, osteophytes and osseous cystic lesions were similar to previously imaged pigs at our institution (Figure 3), so the joints were injected at the discretion of the clinician prior to receiving fluid analysis results (JAH, personal communication). The analysis showed severe non‐degenerative neutrophilic inflammation, and no infectious agents were seen, which is suggestive but not conclusive of non‐infectious arthritis. While degenerative joint disease was the presumed diagnosis at the time of injection, other causes of neutrophilic arthritis such as immune‐mediated arthritis cannot be ruled out. Chronic Erysipelothrix arthritis in pigs has been researched as a model rheumatoid arthritis in humans.^11^ Both diseases show similar pathophysiology such as similar joint histopathology and elevated nucleated cell counts. Pigs have been shown to have elevated cell counts long after evidence of active infection remains.^12^ Whether this is the reason for elevated cell counts in this joint or it is a representation of chronic degenerative joint disease cannot be determined. Synovial fluid analysis is not performed commonly in pigs, and there is no published data on synovial fluid analysis in pigs with various joint pathologies. Given that cell counts are higher in normal joints compared to other species,^6^ pigs may exhibit higher cell counts than expected for a non‐infectious disease, explaining the markedly elevated cell count demonstrated in this study. Despite the advanced pathology in the joint and severe inflammation, the pig responded well to injections and was still showing improvement at follow‐up, 10 months after the injection.

The second synovial fluid analysis, from an elbow which had previously received multiple IA injections in both elbows, had a normal cytology and fluid analysis despite advanced osteoarthritic changes present on CT, which may suggest that the inflammation was successfully treated using corticosteroids and there may be another reason for the return to lameness and diminishing efficacy of the injections in this pig. However, no synovial fluid analysis was performed prior to injections. Due to the limited number of synovial fluid analyses performed, limited conclusions can be made from these data but represent an important future direction for further understanding the pathophysiology of pigs with advanced joint disease.

The main limitations of this study include its retrospective nature, limited number of cases available, and the reliance on client questionnaires to assess patient outcomes. Additionally, the lack of standardized protocols for follow‐up assessments (e.g., lameness scoring and owner reports) may have affected the consistency of outcome reporting. As a referral institution, many of these pet pigs were only evaluated once at the time of injections and follow up visits occurred when the lameness recurred. This prevented lameness evaluation of these pigs while the lameness was improved following injections. A lameness scoring system in production pigs has been developed that uses various behavior, posture, and gait metrics within the scoring system.^13^, ^14^, ^15^ Adopting a scoring system to assess lameness in pet pigs is warranted and would allow for improved assessment of the response to treatment. Recall bias is also a major limitation as clients may have a perception of an improved outcome following the injection. Additionally, clients may not recall whether other treatments or therapies were used that could have contributed to their improvement. Another limitation is the inclusion of all joint types, each with varying pathologies; however, the majority of joints treated were the elbow. The effectiveness of IA injections may vary depending on the joint involved, and further studies should consider focusing on specific joints to determine if certain joints respond better to treatment than others. Additionally, without a matched control group, the true efficacy of the treatment cannot be determined, as some pigs may have experienced spontaneous improvement while others may have had coexisting treatments that contributed to their recovery. Subjectively, it is the authors' experience that pigs tend to respond better to the combination of both systemic NSAID and IA injections with corticosteroids. Future investigation should involve a placebo controlled, blinded study to investigate the benefits of IA injections for treatment of OA in pet pigs.

In conclusion, this study supports the finding that IA injections provide benefit to pet pigs with lameness due to OA in decreasing lameness. While no adverse events were recorded, the sample size was small so further investigation is required to determine the safety of these injections. The main benefit of performing CT examination in these patients is the increased accuracy in diagnosis of the specific joints and disease processes affecting them, which allows for the most effective treatment modality. However, given the small sample size and variability in follow‐up, additional research with larger cohorts and more standardized protocols is needed to determine if there are benefits of using CT guidance and to explore the long‐term effectiveness of IA injections in managing chronic lameness in pigs. This study supports the use of IA injections as a valuable tool in the management of lameness in pet pigs.

## AUTHOR CONTRIBUTIONS

Schultz RH, DVM: Designed the study, collected the data from the medical record, conducted owner surveys, and wrote the manuscript. Hermida JA, DVM, DACVS (Large Animal): Assisted with study design and writing and editing of the manuscript. Hawkins JF, DVM, DACVS: Assisted with manuscript writing and editing.

## FUNDING INFORMATION

No financial support was received for this study.

## CONFLICT OF INTEREST STATEMENT

The authors have no conflicts of interest to disclose.

## Supporting information


**Data S1:** Supporting Information.

## Data Availability

The data is available for review upon request from the corresponding author.
