# Supplementary material for: Diagnostic imaging findings and management of osteoarthritis in pigs with image‐guided intra‐articular injections
Source: Vet Surg. 2025 Aug 27;54(7):1433–42. doi: 10.1111/vsu.70002 (PMC12528815; doi:10.1111/vsu.70002)
Supplement: Supplementary file 1 — Data S1: Supporting Information. [file VSU-54-1433-s001.docx]

Client questionnaire

- How long was (name) lame prior to injections?
- On a scale of 1-5, how would you grade the severity of lameness prior to injections? 1 being no visible lameness, 5 being non-weight bearing and/or unwilling to move
- Following the injections was the lameness: Worse, no different, slightly improved or significantly improved
- On a scale of 1-5, how would you rate the lameness at its best following the injection, using the same scale as previously?
- How long after the injections was any effect seen? 1-3 days, 4-7 days, 1-2 weeks, longer
- How long after the injections was the greatest effect seen? 1-3 days, 4-7 days, 1-2 weeks, longer
- Other than lameness, did you see improvements in attitude or activity levels following the joint injection?
- How long were the improvements apparent? (few weeks, several weeks, several months)
- When the lameness returned, was the severity similar to the initial presentation?
- Was (name) on any other medications for lameness at the time of injection? Were they decreased or discontinued after injections?
- Were there any complications or adverse effects seen? If so, what were they
- Has the joint(s) been treated again here or elsewhere?
- How long was the interval between the initial injection and follow up injections?
- Was there a similar response after repeated injections?
- How long did the effect of follow up injections last?
- Overall, what is your satisfaction with the use of injections to treat joint disease in (name)? Very dissatisfied, Slightly dissatisfied, Neutral, Slight satisfied, Very satisfied
- Would you recommend joint injections for the treatment of joint disease to other pig owners for the treatment of joint disease?
